# Large‐Scale Traveling Atmospheric and Ionospheric Disturbances Observed in GUVI With Multi‐Instrument Validations

**DOI:** 10.1029/2022GL099901

**Published:** 2022-08-25

**Authors:** Katrina Bossert, Larry J. Paxton, Tomoko Matsuo, Larisa Goncharenko, Komal Kumari, Mark Conde

**Affiliations:** ^1^ School of Earth and Space Exploration Arizona State University Tempe AZ USA; ^2^ School of Mathematical and Statistical Sciences Arizona State University Tempe AZ USA; ^3^ Applied Physics Laboratory Johns Hopkins University Laurel MD USA; ^4^ Department of Aerospace Engineering Sciences University of Colorado Boulder CO USA; ^5^ Haystack Observatory Massachusetts Institute of Technology Westford MA USA; ^6^ Geophysical Institute University of Alaska Fairbanks AK USA

**Keywords:** gravity waves, measurements, thermosphere dynamics, traveling ionospheric disturbance

## Abstract

This study presents multi‐instrument observations of persistent large‐scale traveling ionosphere/atmospheric disturbances (LSTIDs/LSTADs) observed during moderately increased auroral electrojet activity and a sudden stratospheric warming in the polar winter hemisphere. The Global Ultraviolet Imager (GUVI), Gravity field and steady‐state Ocean Circulation Explorer, Scanning Doppler Imaging Fabry–Perot Interferometers, and the Poker Flat Incoherent Scatter Radar are used to demonstrate the presence of LSTIDs/LSTADs between 19 UT and 5 UT on 18–19 January 2013 over the Alaska region down to lower midlatitudes. This study showcases the first use of GUVI for the study of LSTADs. These novel GUVI observations demonstrate the potential for the GUVI far ultraviolet emissions to be used for global‐scale studies of waves and atmospheric disturbances in the thermosphere, a region lacking in long‐term global measurements. These observations typify changes in the radiance from around 140 to 180 km, opening a new window into the behavior of the thermosphere.

## Key Points

- For the first time, Global Ultraviolet Imager observations of large‐scale traveling ionosphere disturbances (LSTIDs) in far ultraviolet emissions are presented and validated
- Persistent LSTID/large‐scale traveling atmospheric disturbances (LSTADs) are observed over several hours coinciding with moderate geomagnetic disturbances and a major sudden stratospheric warming (SSW)
- Along‐track horizontal wavelengths of the atmospheric disturbances are ∼2,000–2,500 km and periods are ∼2–2.5 hr

## 1 Introduction

Disturbances in the atmosphere, referred to as traveling atmospheric disturbances (TADs) in the neutral atmosphere and traveling ionospheric disturbances (TIDs) in the ionosphere perturb densities, winds, and temperatures. TIDs/TADs are commonly associated with atmospheric gravity waves (GWs) that originate in the lower and middle atmosphere (Azeem et al., 2015; Negale et al., 2018; Nicolls et al., 2014) and play an important role in thermospheric dynamics (Becker & Vadas, 2020; Lilienthal et al., 2020; Miyoshi et al., 2018; Vadas et al., 2014). Despite the importance of thermospheric GWs, limited observations exist due to the lack of measurements in the thermosphere region.

Large‐scale TIDs (LSTIDs), TIDs with horizontal wavelengths >1,000 km, have been associated with GWs generated due to ion‐drag forcing as well as auroral particle and Joule heating elevated during geomagnetic storms at higher latitudes (Gardner & Schunk, 2010; Hedin & Mayr, 1987; Lyons et al., 2019; Nicolls et al., 2012; Richmond, 1978; S.‐R. Zhang et al., 2019). LSTIDs have also been observed to be correlated with the auroral electrojet indices (Frissell et al., 2022; Hajkowicz, 1991). LSTIDs/large‐scale TADs (LSTADs) have also been observed even during geomagnetically quiet times at smaller amplitudes (Bruinsma & Forbes, 2008; Hedin & Mayr, 1987). Vadas and Liu (2009) demonstrated the potential for large‐scale GWs in the thermosphere to be generated from tropospheric convections at midlatitudes. A number of satellite measurements have been used to demonstrate the presence of large‐scale GWs during geomagnetically quiet times. Trinh et al. (2018) found a correlation between GWs in the stratosphere with GWs observed in Gravity field and steady‐state Ocean Circulation Explorer (GOCE) and CHAMP, indicating vertical coupling between the stratosphere and thermosphere. Vadas et al. (2019) presented observations of medium‐ and large‐scale GWs over the Andes during strong mountain wave activity and low geomagnetic activity using GOCE densities and cross‐track winds. England et al. (2021) showed the presence of a large‐scale GW using GOLD far ultraviolet (FUV) radiances during quiet solar conditions. These observations further contribute to the duality of forcing of LSTIDs/LSTADs from both below and above.

Measurements of waves in the thermosphere remain limited. The Global Ultraviolet Imager (GUVI) instrument onboard the Thermosphere Ionosphere Mesosphere Energetics and Dynamics (TIMED) satellite has been collecting data in orbit since 2002 (L. J. Paxton et al., 2004, 2017). Existing dayside GUVI FUV measurements (115–180 nm) offer the potential for global dayside coverage of perturbations associated with waves in the thermosphere, and an opportunity for coincident measurements with ground‐based and satellite‐based data sets, expanding the limited existing measurements in the thermosphere. This paper reports persistent LSTID/LSTADs observed in multiple instruments between 19 UT 18 January and 5 UT 19 January 2013. These observations include the first measurements by GUVI of LSTADs. The GUVI observations are further substantiated by density and cross‐track wind measurements from GOCE, wind measurements from the Scanning Doppler Imaging Fabry–Perot Interferometers (SDIs) at Poker Flat and Toolik Lake, and the Poker Flat Incoherent Scatter Radar (ISR).

## 2 Observations

This study uses satellite measurements to demonstrate the presence of TADs over a spatial range and ground‐based instrumentation to determine the associated period of persistent TIDs/TADs. A focus was on January 2013 during magnetically quiet (Kp < 4) times in the Northern Hemisphere region near Poker Flat, AK given the availability of concurrent data during this time from GUVI, GOCE, Poker Flat Incoherent Scatter Radar (PFISR), and the SDI. The strongest LSTAD observed in GUVI during this period was on 18 January 2013. The observations from this notable event are discussed in depth in the following sections.

### 2.1 Global Ultraviolet Imager

**Figure 1:**
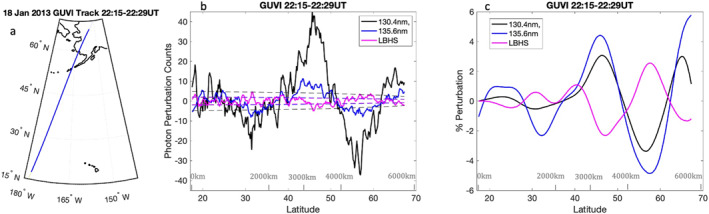
(a) The pierce point latitude and longitude along the Global Ultraviolet Imager (GUVI) path for emissions near 140 km. (b) The residual perturbations in photon counts after using a Savitzky–Golay filter to detrend the data (solid lines) and the noise floor determined from background photon counts (dotted lines). (c) The percent perturbation for each emission filtered for along‐track wavelengths >1,600 km.

The GUVI instrument L1B spectrograph data product provides five wavelength ranges in the FUV including HI 121 nm, OI 130.4 nm, OI 135.6 nm, and the N_2_ Lyman–Birge–Hopfield (LBH) bands divided into shorter LBHS 140–150 nm and longer LBHL 165–180 nm wavelengths (Christensen et al., 2003; L. J. Paxton et al., 1999). In 2007, the instrument stopped scanning spatially and now operates in spectrograph mode, looking at one direction offset from nadir by 47°. In spectrograph mode, 14 pixels record the spectrum of FUV emissions (115–180 nm) over a range of 100 km along track every 3 s (L. J. Paxton et al., 2017). The emissions used in this study are averaged over these 14 pixels. To obtain perturbations associated with these emissions, a pass over Poker Flat, Alaska, is detrended using a third‐order Savitzky–Golay filter over the range of 18°N–67°N. Perturbations were most visible north of 30°N, and GOCE data are not available below 18°N (discussed in next session), so a cutoff of 18°N was used for this study. The third‐order Savitzky–Golay filter for background removal was used to account for the shape of background emissions, which change nonlinearly with solar incidence versus longitude. To eliminate perturbations that may be associated with energetic particle precipitation, emissions near the center of the auroral oval are not used in this study despite the quiet conditions, thus data north of 67°N are excluded. Figure 1a shows the track along the measurement, which took place from 22:15 UT to 22:29 UT on 18 January 2013. Figure 1b shows along‐track perturbations in photon counts for the O 130.4 nm, O 135.6 nm, and LBHS emissions. The dotted line in Figure 1b denotes the background noise based on background photon counts for each respective emission. For photon noise, the SNR = $\sqrt{N}$, where *N* denotes the number of photons. This SNR is further increased by averaging over a number of data points. The background noise shown by the dotted line is thus calculated as $\sqrt{N}/\sqrt{\#\;\text{data}\;\text{points}}$. A running average was applied to the data points using five data points along track (∼100 km along track), which increases the SNR >1 for the faint LBHS perturbations. While this provides a resolution of ∼100 km per data point for this study, it is noted here averaging can be changed for different events. The running average was applied to the perturbations after background subtraction due to the longitudinally changing background signal, which is dependent on solar radiation. Figure 1c shows a plot of these perturbations filtered for along‐track wavelengths of >1,600 km. These filtered perturbations demonstrate the presence of a wave with an along‐track wavelength of ∼2,000 km. The perturbations are most visible in all three emissions from 40° to 60°N.

The measurements from GUVI show both the atomic oxygen 130.4 and 135.6 nm emissions were nearly in phase. The emission from 130.4 nm was stronger than 135.6 nm. However, the percent perturbation for 135.6 nm was ∼20%–30% larger than that of 130.4 nm. While both 130.4 and 135.6 nm emission come from atomic oxygen, 130.4 nm is an allowed transition in atomic oxygen (e.g., L. Paxton & Anderson, 1992) leading to very large optical depths near line center when viewed from above the emitting layer and is created by photoelectron impact excitation and solar resonance scattering. As detailed in Meier (1991), the photons have a low probability of escape near line center. This means that the 130.4 nm emissions come from a broader range of altitudes than the purely photoelectron‐impact‐excited 135.6 nm emissions. These emissions from 130.4 to 135.6 nm appear in phase, thus consistent with the long vertical wavelengths associated with GWs in the thermosphere (Miyoshi & Fujiwara, 2009; Vadas, 2007). The perturbations were also observed in LBHS emissions. While the perturbations would also be expected to be observed in the LBHL emission, the signal in this channel was too weak to retrieve perturbations above the background noise floor for the given averaging. LBH emissions are expected to come from similar altitudes as the 135.6 nm emissions, with a peak emission between 140 and 180 km (England et al., 2021; Meier, 1991; Meier & Lee, 1982; Strickland et al., 1995). The LBHS perturbation was out of phase with 135.6 nm emissions, which agrees with previous GOLD measurements, and has been discussed in England et al. (2021). In the case presented here, the percent perturbation between 40° and 60°N ranged between ∼2% and 5% in the filtered emission data. While this is notably larger than perturbations observed in England et al. (2021), this particular event was also significant in the range of dates for January 2013.

### 2.2 GOCE

**Figure 2:**
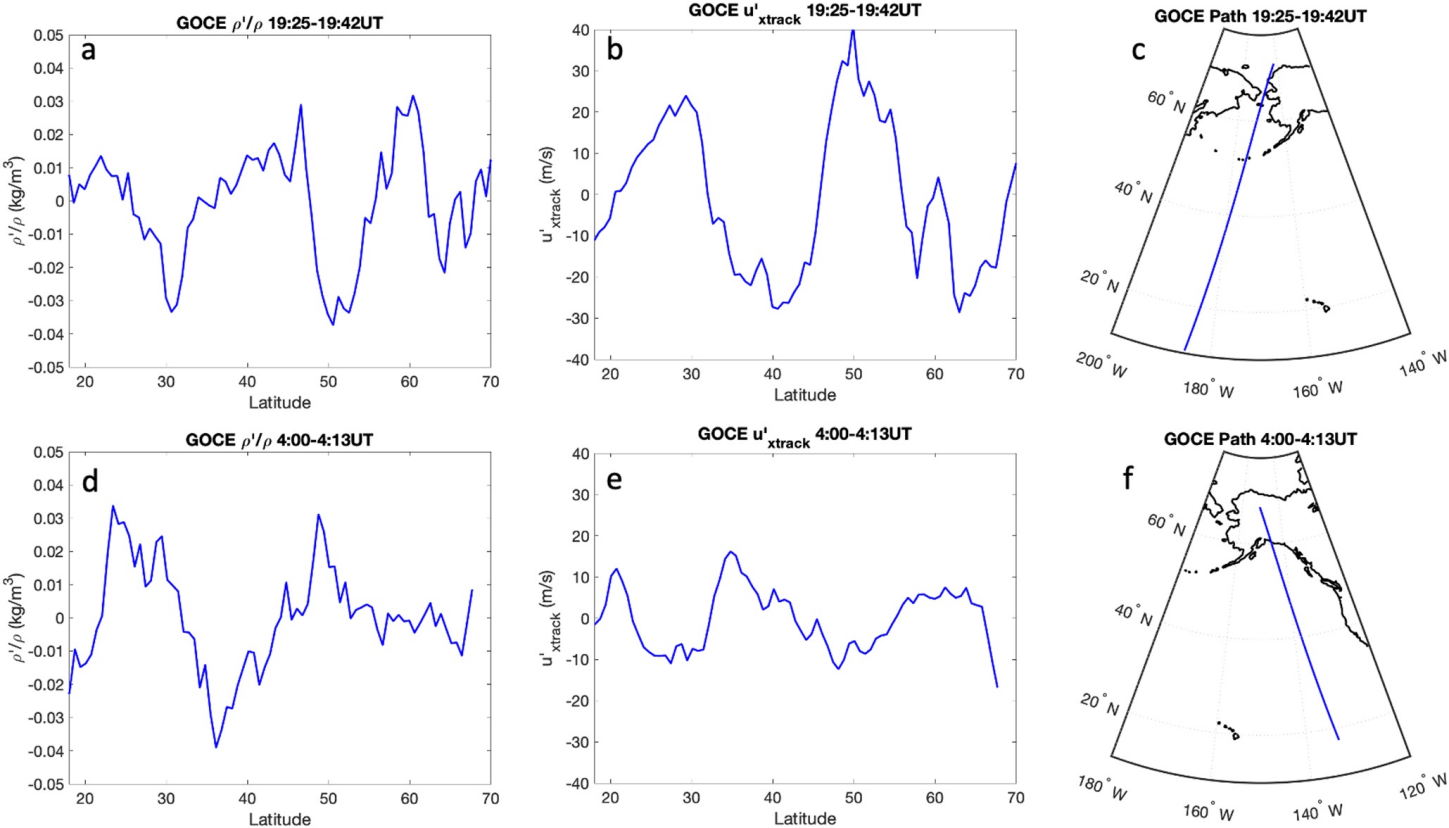
(a–c) Data from the first nearby Gravity field and steady‐state Ocean Circulation Explorer (GOCE) pass and (d–f) data from the second nearby GOCE pass. (a, d) Density perturbations as $\rho^{\prime }/\bar{\rho }$. (b, e) ${u^{\prime }}_{\text{xtrack}}$ derived from cross‐track winds. (c, f) The path of GOCE during the measurements.

Several hours before and after the GUVI pass over Alaska, the GOCE satellite has passed over Alaska. Both passes demonstrate the presence of LSTADs in densities and cross‐track winds. The use of density measurements to study medium‐scale GWs has previously been done with both CHAMP (Bruinsma & Forbes, 2008) and GOCE (Trinh et al., 2018). Additionally, GOCE has been used to detect the presence of both medium‐scale and large‐scale GWs during geomagnetically quiet times (Vadas et al., 2019). The GOCE satellite collected density and cross‐track wind data at 250 km in altitude during the time period of this study. The data shown in Figure 2 were detrended using a third‐order polynomial Savitzky–Golay filter over the range of the data set. Data at latitudes below 18°N were not used in this study due to the eclipse transition and potential impacts on cross‐track wind measurements. Figures 2a–2c show data from 19:25 UT to 19:42 UT, and Figures 2d–2f show data from 4:00 to 4:13 UT. Both time periods show the presence of perturbations in mass densities and cross‐track winds. At both times, mass density perturbation amplitudes were ∼3%–4% of the background. Cross‐track wind perturbations were about twice as large in the earlier pass. This may be due to TAD amplitude or the orientation of the satellite path relative to the wave itself. During both passes, perturbations were seen further south than in the GUVI measurements. Along‐track horizontal wavelengths were ∼2,000 km near 19:30 UT and ∼2,500 km near 4:00 UT, which were similar to those that GUVI measured. However, given the time differences between these measurements and the GUVI measurements, it is unlikely that these are the same waves. We note here that both instruments measured similar horizontal along‐track scales.

### 2.3 SDI

**Figure 3:**
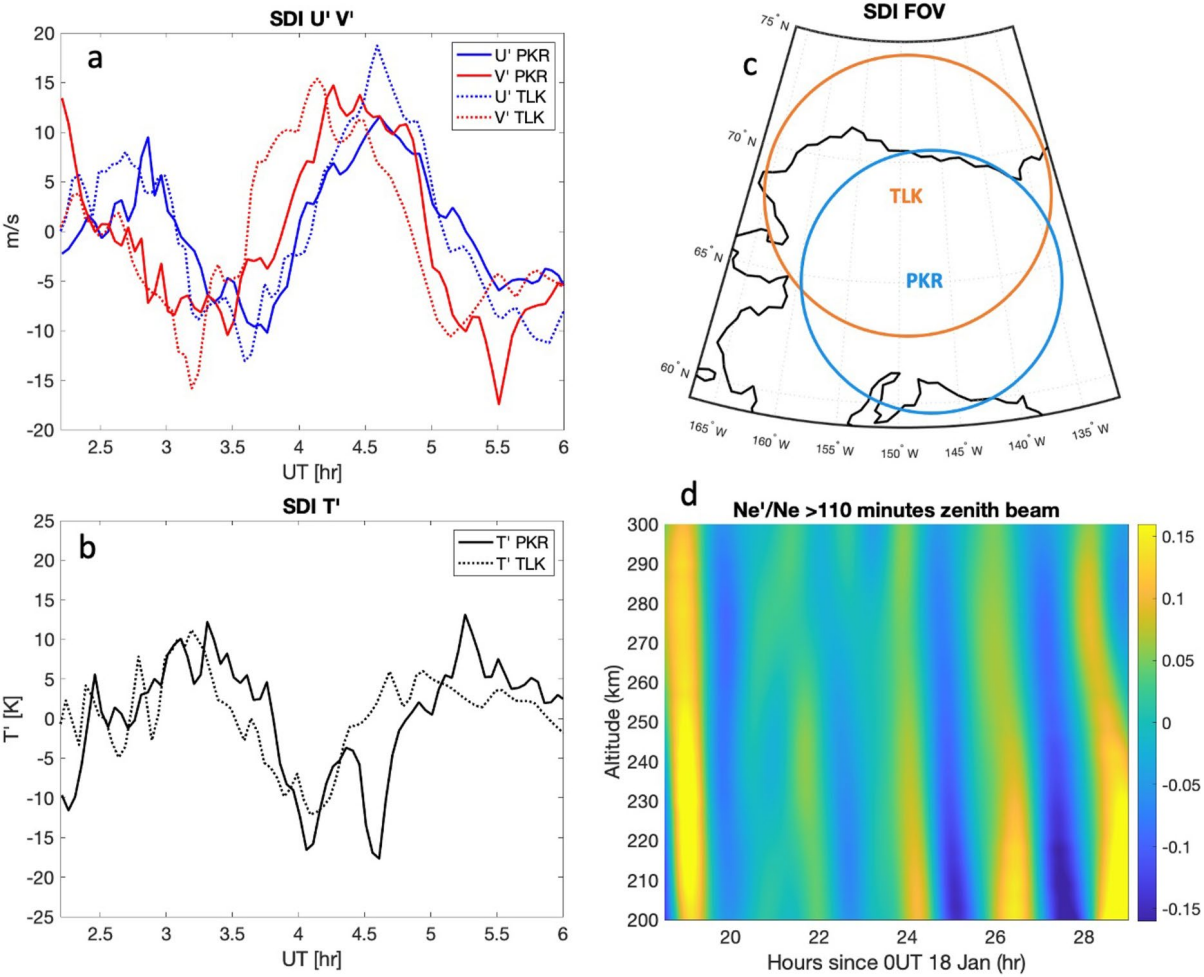
(a) Derived U′ in blue and V′ in red, with Poker Flat (PKR) in solid lines and Toolik Lake (TLK) in dotted lines. (b) T′ from PKR with a solid black line and T′ from TLK with a dotted black line. (c) The field of view (FOV) over which winds and temperatures were averaged in each instrument (Toolik Lake: orange; Poker Flat: blue). (d) Electron density perturbations, Ne/Ne′, filtered for periods >110 min observed by the Poker Flat Incoherent Scatter Radar (ISR) from 18.5 UT on 18 January to 5 UT on 19 January indicating the presence of 2–2.5 hr period large‐scale traveling ionosphere disturbances (LSTIDs) with large vertical wavelengths.

The SDIs at Poker Flat (65.1°N, 147.5°W) and Toolik Lake (68.6°N, 149.3°W) have been used to measure temperatures, T, zonal winds, U, and meridional winds, V, over Alaska near ∼250 km in altitude using the 630 nm emission. This emission layer itself extends below 200 km and above 300 km in altitude and can vary based on background conditions (Ogawa et al., 2002), thus is most sensitive to long vertical wavelengths. The SDI data consist of 115 different look regions in the sky, retrieving U and V wind components (Conde & Smith, 1995, 1998; Itani & Conde, 2021). For this study of LSTAD perturbations, data from 19 January were used starting at ∼2 UT. Measured T, U, and V were averaged over the field of view (FOV) ∼900 km in diameter for both the Poker Flat and Toolik Lake SDIs. Look regions within one degree of the zenith were not used for derived U and V wind components in the average due to larger associated uncertainty from the small horizontal component of the wind vector. U, V, and T are detrended using a Savitzky–Golay filter to retrieve perturbations. Figure 3a shows the derived perturbations for U′ and V′, and Figure 3b shows T′. Figure 3c shows the spatial extent of these measurements. An approximately 2–2.5 hr period perturbation was observed in U′, V′, and T′ over Toolik Lake and Poker Flat. Both instruments showed a phase shift between U′, V′, and T′, with the largest shift between wind and temperature perturbations. These waves persisted from the initial time of SDI measurements just after 2–6 UT. Wind perturbations in both U and V were ∼10–15 m/s over the averaged area, and T′ was ∼15 K. The GOCE satellite measurements overlapped with this region near 4:10 UT. During this time, the SDI observed V′ between 5 and 10 m/s, and U′ between 0 and 5 m/s for perturbations averaged over the FOV of the SDI. The GOCE cross‐track wind perturbation was ∼10 m/s in this region.

### 2.4 PFISR

ISR has been used extensively to study both MSTIDs and LSTIDs (Frissell et al., 2022; Kirchengast et al., 1996; Negale et al., 2018; Nicolls et al., 2014; Panasenko et al., 2018). During the time period discussed in this paper, the PFISR was operating in long pulse mode, allowing for retrieval of electron densities, Ne, during the 18–19 January 2013 period. The methods discussed in Negale et al. (2018) are used to retrieve Ne′/Ne. Figure 3d shows Ne′/Ne from 200 to 300 km in altitude filtered for periods >110 min and <4 hr. During this time period from 18.5 UT on 18 January to 5 UT on 19 January, LSTIDs were persistent with periods ranging from 2 to 3 hr. The periods observed in ISR measurements align with the SDI measurements in the same time period. The ISR also observed LSTIDs during the times of GUVI and GOCE passes over the region. While the ISR measurements overlap in altitude with the SDI and GOCE measurements, GUVI FUV emissions peak below the region of ISR measurements. However, the downward phase progression observed in the ISR indicates that these TIDs were propagating upward from a lower altitude than 200 km, and the associated long vertical wavelengths would contribute to larger GUVI emissions due to lack of cancelation over the emission layer that would otherwise occur with shorter vertical wavelengths. This would especially be the case from the top side of the emission peaks, where long vertical wavelengths ($\lambda_{z}$ > 100 km) were observed in the ISR at least down to 200 km. The ISR also shows no significant Ne′/Ne growth above 200 km and a notable decrease in Ne′/Ne amplitudes between 240 and 300 km. Near the time of GUVI measurements, between 22 and 23 UT and 200–240 km, Ne′/Ne values are ∼0.05% or 5%. These Ne′/Ne are in line with previous PFISR MSTID measurements which can be as high as 0.2 depending on the time of day and altitude (Negale et al., 2018; Vadas & Nicolls, 2009). While this is a higher altitude than the peak emissions from GUVI, and Ne′/Ne is not necessarily a 1:1 equivalent with density perturbations (Vadas & Nicolls, 2009), the magnitude is in line with observed GUVI FUV perturbations ranging from 2% to 5%. Another aspect of comparing ISR measurements to GOCE and GUVI is that the ISR measurements only give information near the Poker Flat research range (65°N, 147.5°W). At 4 UT, GOCE observed the large perturbations in winds and densities between 20° and 50°N. GUVI observed large perturbations between 40°N and 65°N near 22:30 UT. Winds over this meridional range of >1,000 km may change significantly, resulting in amplitude growth or dissipation of the observed waves in the thermosphere as they propagate. The ISR does confirm the presence of LSTIDs that are persistent over the times of satellite‐based observations.

## 3 Discussion

The combined measurements over this period all demonstrate the presence of LSTIDs/LSTADs. While GUVI measurements do not necessarily overlap the GOCE and SDI measurements, the PFISR demonstrates that the observed LSTIDs are observed as LSTADs in all three other instruments. This combination of measurements strongly suggests that LSTADs are detected in GUVI FUV emissions. This particular event appears relatively large in amplitude for the small time period studied here, and perturbations are detected by GUVI in the 130.4, 135.6, and 140–150 nm FUV emissions. It is likely that smaller amplitude events can be seen, especially in the 130.4 and 135.6 nm emissions. For this event, the SNR for 130.4 nm was ∼10 and for 135.6 nm was ∼5. Additionally, only five along‐track points were used in a moving average 100 km along track, and this averaging could be increased for smaller amplitude events. We note that larger amplitude events are also possible.

**Figure 4:**
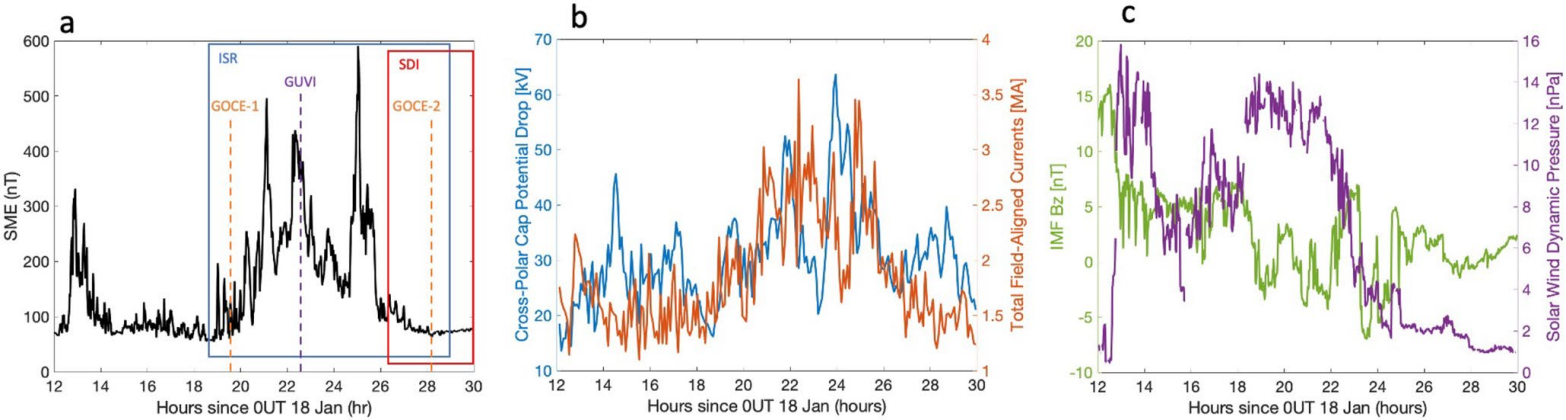
(a) An overlap of the SuperMAG Electrojet (SME) index with measurement times of instruments used in this study. (b) The cross‐polar cap potential drop (blue) overplotted with total field aligned currents (red). (c) The solar wind dynamic pressure (purple) and interplanetary magnetic field Bz component (green) over the same period.

This event demonstrates the importance of recognizing GW sources from above and below in the thermosphere region, and the need for further studies of multisource wave forcing in the thermosphere affecting day‐to‐day variability. There are two potential sources of disturbances, which include (a) the geomagnetic activity at high latitudes induced by the sustained compression of the magnetosphere by the elevated solar wind dynamic pressure and/or (b) the sudden stratospheric warming (SSW) overlapping the same time period. The SuperMAG Electrojet (SME) index (Newell & Gjerloev, 2011a, 2011b) has previously been used to show auroral activity as a driver of LSTID generation (Frissell et al., 2022). Liou et al. (2007) have shown the evidence of prompt and sustained auroral particle precipitation associated with strong plasma flows on the dayside in response to the elevated solar wind dynamic pressure using DMSP and POLAR data, referring to the phenomena as “compression aurora.” Additionally, Cherniak and Zakharenkova (2018) showed increases in field aligned currents (FACs) are associated with LSTIDs. Figure 4a shows a plot of the SME overplotted with measurement time windows of instruments used in this study from 12 UT on 18 January to 6 UT on 19 January. The SME increases starting near 19 UT on 18 January and decreases by 4 UT on 19 January. During this time, increases in the cross‐polar cap potential (CPCP) drop and FACs are also observed, and this is shown in Figure 4b. The CPCP and FACs were computed by the Assimilative Mapping of Geospace Observations (AMGeO; Matsuo, 2020) from SuperDARN plasma drifts (Greenwald et al., 1995), ground magnetic field perturbations distributed by the SuperMAG data service (Gjerloev, 2012), and iridium magnetic field perturbations processed and distributed by the AMPERE program (Anderson et al., 2000). Figure 4c shows the sudden increase in solar wind dynamic pressure over13–15 UT and again over 18–22 UT, resulting in an expected compression of the magnetosphere, elevated FACs, and ionospheric currents and likely enhanced auroral particle precipitation. The interplanetary magnetic field (IMF) Bz shown in Figure 4c suggests that moderately elevated geomagnetic activity during the period is not necessarily associated with the southward turning of IMF Bz, as Bz remains mostly positive with small fluctuations between positive and negative values. The data shown in Figure 4 suggest that both ion drag and Joule heating may be a potential forcing mechanism for the observed LSTIDs/LSTADs. It is important to note that LSTIDs generated by the auroral electrojet are not necessarily generated over Alaska and may propagate over thousands of kilometers from their region of generation. We also note that during the time period of these observations, the Kp index is 3, which is indicative of relatively low auroral energy inputs (e.g., Y. Zhang & Paxton, 2008). The observation period is largely on the dayside and partially during the early evening after sunset. There were minimal disturbances in the magnetic field as measured by ground magnetometers near Poker Flat, AK during the period of ISR observations.

In addition to the increased SME index, FACs, CPCPs, and solar wind, these observations also overlap with a SSW event (Coy & Pawson, 2015; Nath et al., 2016). While SSWs may suppress orographic GWs (Triplett et al., 2017) and have been associated with decreased MSTID activity (Frissell et al., 2016), disturbances to the polar vortex have also been linked to the generation of GWs (Becker et al., 2022; Bossert et al., 2020). Given the activity in the stratosphere, forcing from below cannot definitively be ruled out as a potential source. Additionally, large‐scale secondary GW generation from either stratospheric generated waves or secondary GWs generated from breaking GWs originating from the auroral electrojet may also be potential sources. Observational evidence during this event study reinforces the importance of recognizing GW sources from above and below in the thermosphere region and the need for further studies using multiple measurements to examine the generation mechanisms of multisource waves in the thermosphere that play a key role in transporting momentum and energy from one region to another.

## 4 Summary

The 18–19 January 2013 event analysis constitutes the first report of corroborated observations of LSTADs with GUVI. The LSTADs are coincident with LSTIDs observed in PFISR. Observed horizontal wavelengths from GUVI and GOCE are in the range of 2,000–2,500 km along track. Periods observed with the ISR and SDIs are in the range of 2–2.5 hr. Assuming the along‐track wavelength is close to the horizontal wavelength, phase speeds of these TADs/TIDs would be ∼300 m/s, which is reasonable for GWs in the thermosphere. The ISR data have also demonstrated vertical wavelengths of >100 km and likely dissipation above 250 km in altitude. The waves are observed during a time of increased SME index, FACs, CPCPs, and solar wind dynamic pressure, though the Kp index is only 3. This observation period is also coincident in time with a major SSW. Multiple potential sources of the observed waves exist, demonstrating a need for more measurements in this region. The observations of TADs from the GUVI instrument establish a new source of measurements for understanding waves in the thermosphere and day‐to‐day variability in this region.

## Data Availability

All data used are publicly available, and links to these publicly available data sets are given below: GUVI: http://guvitimed.jhuapl.edu; GOCE: https://earth.esa.int/eogateway/missions/goce/data; SDI: https://sdi/server.gi.alaska.edu/sdiweb/index.asp; PFISR: https://amisr.com/amisr/links/data‐access/; SuperMAG SME index: https://supermag.jhuapl.edu/indices; Solar Wind data from OMNIWeb: https://omniweb.gsfc.nasa.gov. FACs and CPCPs are computed using the AMGeO open source software (https://amgeo.colorado.edu/) from publicly available AMPERE‐Iridium (http://ampere.jhuapl.edu/), SuperDARN (http://vt.superdarn.org/), and SuperMAG (https://supermag.jhuapl.edu/) data.
